# Smoking and Complications After Cancer Surgery: A Systematic Review and Meta-Analysis

**DOI:** 10.1001/jamanetworkopen.2025.0295

**Published:** 2025-03-07

**Authors:** Clement Wong, Siti Khadijah Binti Mohamad Asfia, Paul S. Myles, John Cunningham, Elizabeth M. Greenhalgh, Emma Dean, Sally Doncovio, Lisa Briggs, Nicholas Graves, Nikki McCaffrey

**Affiliations:** 1 Deakin Health Economics, School of Health and Social Development, Institute for Health Transformation, Deakin University, Burwood, Victoria, Australia; 2 Ministry of Women, Early Childhood and Community Wellbeing Development (KPWK), Sarawak, Malaysia; 3 Department of Anaesthesiology and Perioperative Medicine, Alfred Hospital and Monash University, Melbourne, Australia; 4 Neurosciences Institute, Epworth Richmond, Richmond Victoria, Australia; 5 Prevention Division, Cancer Council Victoria; 6 Quit, Cancer Council Victoria; 7 Research & Policy Manager, BreastScreen Victoria, Australia; 8 Thoracic Oncology Group Australasia; 9 Health Services & Systems Research, Duke-NUS Medical School, Singapore; 10 Deakin Health Economics, School of Health and Social Development, Institute for Health Transformation, Deakin University, Geelong, Victoria, Australia; 11 Cancer Council Victoria

## Abstract

**Importance:**

Surgical cancer treatments may be delayed for patients who smoke over concerns for increased risk of complications. Quantifying risks for people who had recently smoked can inform any trade-offs of delaying surgery.

**Objective:**

To investigate the association between smoking status or smoking cessation time and complications after cancer surgery.

**Data Sources:**

Embase, CINAHL, Medline COMPLETE, and Cochrane Library were systematically searched for studies published from January 1, 2000, to August 10, 2023.

**Study Selection:**

Observational and interventional studies comparing the incidence of complications in patients undergoing cancer surgery who do and do not smoke.

**Data Extraction and Synthesis:**

Two reviewers screened results and extracted data according to the Meta-Analyses of Observational Studies in Epidemiology (MOOSE) reporting guidelines. Data were pooled with a random-effects model and adjusted analysis was performed.

**Main Outcomes and Measures:**

The odds ratio (OR) of postoperative complications (of any type) for people who smoke currently vs in the past (4-week preoperative cutoff), currently smoked vs never smoked, and smoked within shorter (2-week cutoff) and longer (1-year cutoff) time frames.

**Results:**

The meta-analyses across 24 studies with a pooled sample of 39 499 participants indicated that smoking within 4 weeks preoperatively was associated with higher odds of postoperative complications compared with ceasing smoking for at least 4 weeks (OR, 1.31 [95% CI, 1.10-1.55]; n = 14 547 [17 studies]) and having never smoked (OR, 2.83 [95% CI, 2.06-3.88]; n = 9726 [14 studies]). Within the shorter term, there was no statistically significant difference in postoperative complications between people who had smoked within 2 weeks preoperatively and those who had stopped between 2 weeks and 3 months in postoperative complications (OR, 1.19 [95% CI, 0.89-1.59]; n = 5341 [10 studies]), although the odds of complications among people who smoked within a year of surgery were higher compared with those who had quit smoking for at least 1 year (OR, 1.13 [95% CI, 1.00-1.29]; N = 31 238 [13 studies]). The results from adjusted analyses were consistent with the key findings.

**Conclusions and Relevance:**

In this systematic review and meta-analysis of smoking cessation and complications after cancer surgery, people with cancer who had stopped smoking for at least 4 weeks before surgery had fewer postoperative complications than those smoking closer to surgery. High quality, intervention-based evidence is needed to identify the optimal cessation period and inform clinicians on the trade-offs of delaying cancer surgery.

## Key Points

**Question:**

What is the association between preoperative smoking status or cessation duration and complications after cancer surgery?

**Findings:**

In this systematic review (n = 54 studies) and meta-analysis (n = 24 studies), patients who had smoked within 4 weeks of cancer surgery had 31% greater odds of postoperative complications vs those who had quit for longer. There was no statistically significant difference between patients who had smoked within 2 weeks of surgery and those who last smoked 2 weeks to 3 months prior.

**Meaning:**

These results suggest that earlier intervention and smoking cessation therapies may reduce the risk of postoperative complications among patients considering cancer surgery.

## Teaser

This systematic review and meta-analysis examines the association between smoking status or smoking cessation time and complications after cancer surgery.

## Introduction

Tobacco smoking is a well-known risk factor for postoperative complications, including in cancer populations.^1,2,3,4^ These complications include increased risk of morbidity, wound issues, surgical site infections, neurological complications, and lung problems.^3,5,6,7^ Moreover, postoperative complications can reduce long-term median survival by up to 70%, accounting for 8 out of 10 surgery-related deaths.^8^ Some clinicians may consider delaying surgery for patients with cancer who smoke,^9,10^ particularly as the risk of complications may be higher due to immunosuppression, delayed wound healing and cancer treatments.^2,11^ For example, 40 percent of recently surveyed thoracic surgeons in the United States (N = 200) required their patients to stop smoking before lung resection, usually at least 2 to 3 weeks before surgery.^12^ However, there is a delicate trade-off with the risk of further disease progression while patients quit.

Quitting smoking before surgery can mitigate some of the smoking-related pathophysiological changes that increase the risk of complications, such as reduced tissue perfusion, impaired inflammatory processes, proliferation of fibroblasts and collagen production.^3,13,14,15^ A number of recently published systematic reviews have investigated the effects of smoking and smoking cessation on surgical complications in conditions such as arthroplasty,^16^ spine surgery,^17^ and foot and ankle surgery.^18^ A recently published overview of systematic review evidence on the effectiveness of preoperative smoking cessation interventions before multiple types of surgery concluded there was high-certainty evidence that interventions including behavioral support and pharmacotherapy started at least 4 weeks before surgery are necessary to prevent postoperative complications.^7^ Several systematic reviews have identified effective strategies for smoking cessation in the cancer setting including providing a tobacco specialist for smoking cessation care, training clinicians,^19^ and behavioral therapy combined with pharmacological interventions.^20,21^ However, to date, no reviews have assessed the association of smoking cessation interventions, smoking status, and cessation timing with postoperative complications for people living with cancer to inform clinical practice and public health policy.

This study aims to quantify the risks of postoperative complications in patients with cancer based on smoking status and history of cessation. A systematic review was conducted to identify and assess interventional and observational studies published since 2000 investigating postoperative complications among patients with cancer who do and do not smoke. Meta-analyses were conducted to quantify the risks for patients living with cancer of postoperative complications among those who currently or recently smoked, compared with those who had quit smoking for longer periods of time and those who had never smoked.

## Methods

This systematic review and meta-analysis follow the Meta-analysis of Observational Studies in Epidemiology (MOOSE) reporting guideline. The protocol was published in the International Prospective Register of Systematic Reviews (PROSPERO), record number CRD42021293195.^22^

### Search Strategy, Selection Criteria, and Screening Process

Embase, Medline Complete, CINAHL, and Cochrane Library databases were searched for articles published since January 1, 2000, meeting the eligibility criteria (eTable 1 in Supplement 1). An initial search was conducted on October 19, 2021, and updated on August 10, 2023 (eTable 2 in Supplement 1).

Initial search results were screened by 2 reviewers (S.A. and N.M.). Following deduplication, the title and abstracts, then full-text articles were screened against the inclusion and exclusion criteria. Backward- and forward-citation tracing was also conducted to identify any relevant studies missed by the search.^23^

For the updated search, Research Screener^24^—a machine learning tool that orders relevant studies—was used to review the titles and abstracts. Two reviewers (C.W. and N.M.) independently screened 50 titles and abstracts ordered by greatest relevance, discussing and resolving any disagreements. One reviewer (C.W.) screened the remaining titles and abstracts in rounds of 50; after each round, Research Screener used the include/exclude screening decisions to update the model and reorder remaining results for greatest likelihood of inclusion. Screening was considered complete when 4 consecutive rounds (200 results in total) contained only excluded studies.

### Data Extraction Process

Data extraction was independently conducted by 2 authors (S.A. and C.W.). Disagreements were discussed and resolved with a third author (N.M.). Information on the following was extracted from each study: smoking status; sample characteristics such as age, sex, cancer type, surgery type, neoadjuvant treatment; intervention; and the incidence of each type of complication. The total count of (unique) individuals with any complication was used as the incidence of overall complications. For studies reporting disaggregated complications only, the maximum count of any single complication type was used to conservatively avoid double counting or overstating overall complications. Estimates from adjusted analyses along with 95% CI or standard errors and approaches to missing data, and incidence of mortality 30 days postsurgery were also extracted where available.

### Risk of Bias

Studies were appraised for risk of bias by 2 authors (C.W. and N.M.) independently, with disagreements discussed to establish consensus. Nonrandomized studies were assessed using the Newcastle-Ottawa scale^25^ and randomized studies were assessed using the Cochrane risk-of-bias tool.^26^

### Statistical Analysis

Analyses were conducted using ReviewManager version 5.4 (RevMan). Estimates were calculated by pooling study-specific odds ratios (ORs); statistical heterogeneity was assessed using the Cochrane *Q*-test and inconsistency across the studies was quantified through the *I*^2^ statistic.^27^ Random-effects models^28^ were conservatively specified a priori for this review (see PROSPERO registration),^22^ due to the anticipated clinical and methodological diversity across the studies with respect to different types of cancer, surgical procedures, and types of postoperative complications.^28^

As categorizations of smoking history differed across the included studies, in the primary analysis current smoking was defined by smoking within 4 weeks of the operation,^2^ past smoking by ceasing 4 weeks or more preoperatively and never smoking by no lifetime smoking. The 4-week threshold was informed by a recent overview of systematic reviews which indicated fewer postoperative complications following noncancer-specific surgical procedures.^7^ For studies that quantified smoking in days per month rather than weeks, 30 days or 1 month was considered equivalent to 4 weeks. To include information from studies that did not strictly differentiate smoking at the 4-week point, sensitivity analyses were conducted using a flexible cutoff for cessation between 4 to 8 weeks before surgery. Consideration of this flexible cutoff arose during data extraction, as it became clear that a stricter definition would exclude estimates from several studies using similar cessation periods of within 6 or 8 weeks.

Smoking cessation timings were also categorized into different groups to compare shorter-term (within 2 weeks or between 2 weeks and 3 months) and longer-term (within or beyond a year) cessation periods. The smoking cessation periods were chosen during data extraction based on the most commonly reported smoking cessation periods across the included studies. Thirty-day mortality and adjusted analyses (adjusted for age, sex, comorbidities such as diabetes or chronic obstructive pulmonary disease, and surgery characteristics) for postoperative complications were also estimated as sensitivity analyses as stated in the systematic review protocol.^22^ Multivariate logistic estimates were pooled to produce an adjusted odds ratio (OR); where available, studies’ descriptions of handling missing data were also recorded.

## Results

### Search and Screening Results

**Figure 1.**
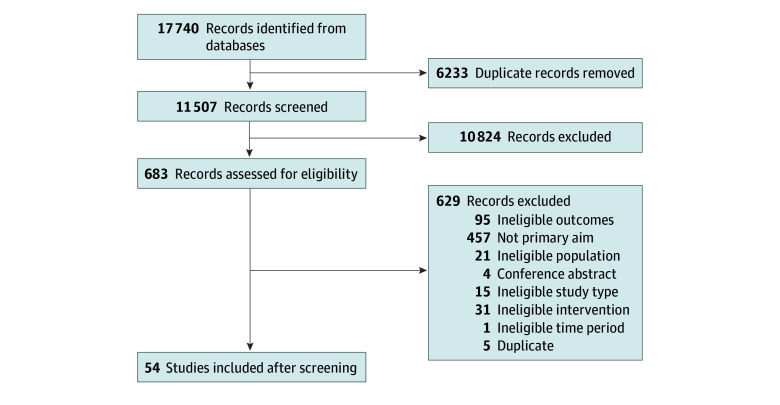
Flow Diagram of Searched, Screened, and Included Studies PRISMA flow diagram of searched, screened, and included studies.

The search strategy identified 17 740 records (initial search, n = 14 656 studies; update search, n = 3084). Among these records, 6233 were duplicates, and 10 824 were excluded after title and abstract screening (Figure 1). Use of Research Screener for the updated search concluded after 15 rounds as there were no relevant abstracts in rounds 12 to 15.

After reviewing 683 full-text articles, 54 studies met the eligibility criteria. Thirty (56%) of the included studies contained sufficient information for inclusion in the meta-analyses, and 17 studies were included in the primary analysis comparing current and past groups.

### Study Characteristics

A summary of the included studies is outlined in the Table. Details for individual studies are provided in eTable 3 in Supplement 2.

**Table.** Summary Characteristics of Included Studies

Characteristic	No. (%)
No.	54
Country	
Australia	1 (1.9)
China	4 (7.4)
Denmark	2 (3.7)
Germany	2 (3.7)
Italy	1 (1.9)
Japan	15 (27.8)
Poland	1 (1.9)
South Korea	3 (5.6)
Spain	1 (1.9)
Taiwan	1 (1.9)
UK	3 (5.6)
US	20 (37.0)
Cancer type	
Bladder	3 (5.6)
Breast	5 (9.3)
Colorectal	5 (9.3)
Esophageal	5 (9.3)
Gastrointestinal	4 (7.4)
Head and neck	4 (7.4)
Liver	1 (1.9)
Lung	27 (50.0)
Pancreatic	1 (1.9)
Prostate	1 (1.9)
Skin	1 (1.9)
Study type	
Cohort	51 (94.4)
Case-control	2 (3.7)
Randomized clinical trial	1 (1.9)
Neoadjuvant therapy	
Included	33 (61.1)
Excluded	8 (14.8)
Not reported	13 (24.1)
Complication type	
Pulmonary	41 (75.9)
Cardiovascular	28 (51.9)
Medical	16 (29.6)
Wound	26 (48.1)
Neurological	17 (31.5)
Renal	10 (18.5)
Genitourinary	12 (22.2)
Thromboembolic and/or vascular	11 (20.4)
Mortality	25 (46.3)
Bleeding	17 (31.5)
Gastrointestinal	6 (11.1)
Anastomotic leak	10 (18.5)
Other	6 (11.1)
Liver	1 (1.9)
Mortality	25 (46.3)

Some included studies involved more than 1 type of cancer or more than 1 type of complication such that totals exceed N = 54.

Most of the 54 studies were conducted in either the US (n = 20)^9,10,29,30,31,32,33,34,35,36,37,38,39,40,41,42,43,44,45^ or Japan (n = 15).^46,47,48,49,50,51,52,53,54,55,56,57,58,59,60^ Lung cancer was the most commonly studied cancer type (n = 27),^9,10,29,31,34,36,39,40,43,46,49,51,52,53,54,55,56,57,61,62,63,64,65,66,67,68^ and only 2 studies^35,48^ included patients across different types of cancer. Almost all studies involved resection surgical procedures or breast reconstruction, although some studies spanned more varied surgical procedures.^35,38,56^ There was 1 intervention-based study conducting a randomized clinical trial (RCT),^69^ and 2 case-control studies,^44,66^ with all other studies using an observational design.^9,10,29,30,31,32,33,34,35,36,37,38,39,40,41,42,43,45,46,47,48,49,50,51,52,53,54,55,56,57,58,59,60,61,62,63,64,65,67,68,70,71,72,73,74,75,76,77,78,79,80^

Approaches toward reporting, inclusion, and exclusion of neoadjuvant therapy differed. Approximately a quarter of the included studies (n = 13) did not report clearly on neoadjuvant therapy or preoperative treatments.^30,42,44,46,49,52,55,64,65,66,75,77,78^ While most studies (n = 33) included patients that received some form of preoperative therapy (eg, chemotherapy, radiation therapy, or immunotherapy), 5 of these did not report the prevalence of these therapies among their sample.^29,32,37,54,62^ Furthermore, 8 studies explicitly excluded patients with neoadjuvant and/or preoperative therapy from their analysis.^39,48,51,53,57,68,69,72^

Most of the included studies examined more than 1 type of postoperative complication. The majority (76% [n = 41]) measured some form of pulmonary complication, with cardiovascular (52% [n = 28]) or wound complications (48% [n = 26]) and mortality outcomes (46% [n = 25]) also commonly reported.

### Risk of Bias

The Newcastle-Ottawa scale scores for the 53 observational studies are summarized in eTable 4 in Supplement 2. Forty-four studies (83%) were high quality (7-9 stars). Areas where studies tended to score lower were in the (1) assessment of outcome and (2) follow-up long enough for the outcome to occur criteria. For the former, some studies indicated that the author/research team was responsible for determining if a postoperative complication occurred without asserting that these outcomes were independently established. For the latter, studies did not clearly define a timeframe during which postoperative complications developed.

The study by Thomsen and colleagues^69^ was assessed with the Cochrane risk-of-bias tool and presented some concerns for the effect of assignment to intervention. All other domains for this study received low risk of bias judgments.

### Meta-Analysis Results

**Figure 2.**
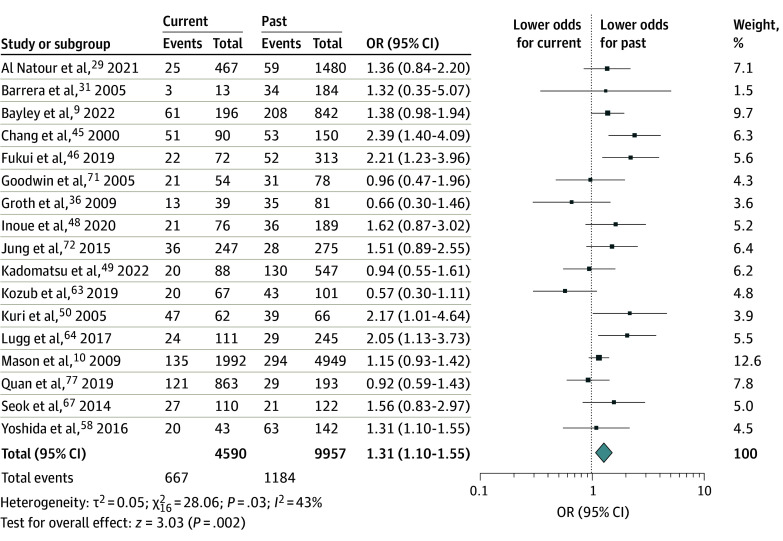
Postoperative Complications Between Current and Past Smoking Unadjusted odds ratio (OR) of any postoperative complication between current (last smoked within 4 weeks before surgery) and past (last smoked beyond 4 weeks before surgery) smoking.

**Figure 3.**
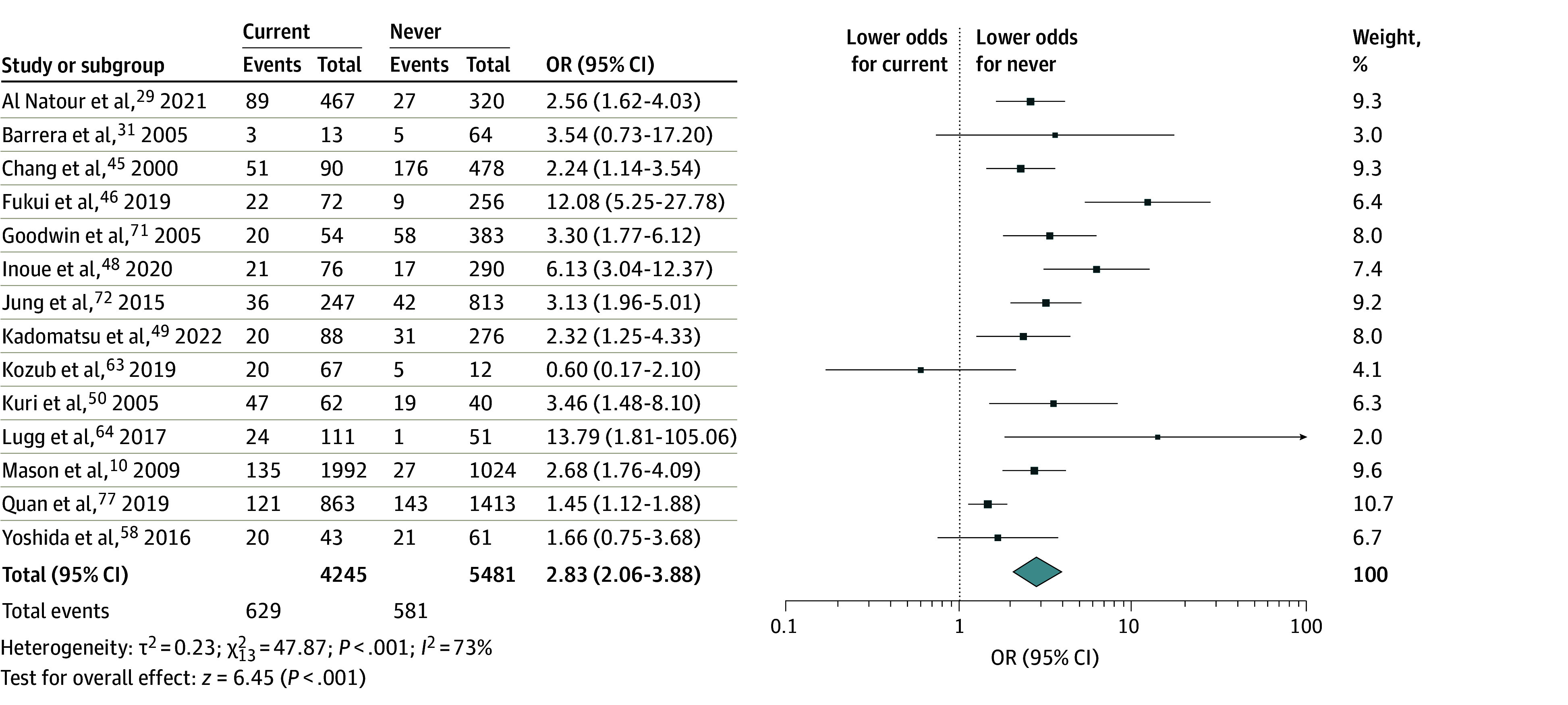
Postoperative Complications Between Current and Never Smoking Unadjusted odds ratio (OR) of any postoperative complication between current (last smoked within 4 weeks before surgery) and never smoking.

**Figure 4.**
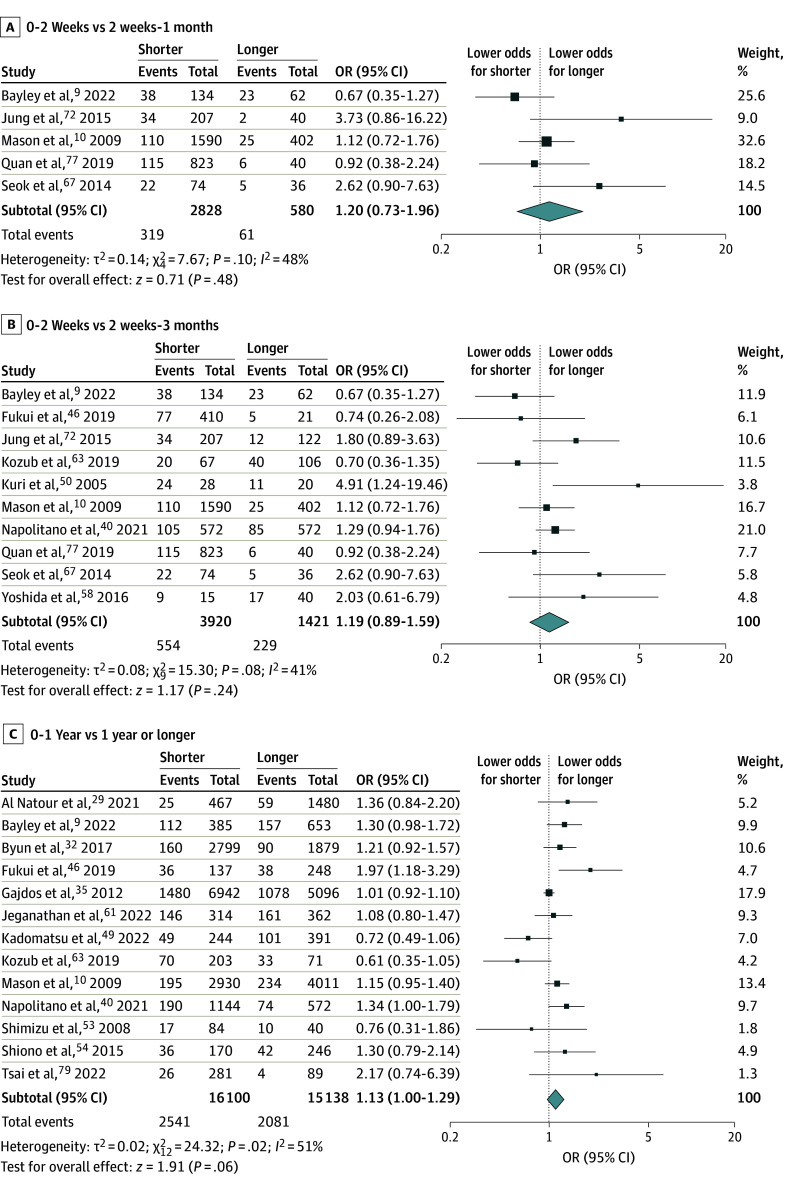
Postoperative Complications by Cessation Timing Unadjusted odds ratio (OR) of any postoperative complication by period of last smoked cigarette.

The primary analyses from the random-effects models are presented in Figures 2, 3, and 4. The sensitivity analyses with flexible cutoffs for smoking cessation between 4 to 8 weeks, cancer type disaggregation, and adjusted analyses are provided in eFigures 1, 2, 3, 4, 5, and 6 in Supplement 1 as the findings are similar. Of note, the adjusted analyses used the flexible cutoff between 4 and 8 weeks as there were very few studies using the stricter primary definition of a 4-week threshold.

### Postoperative Complications by Groups

The meta-analysis compares the incidence of any postoperative complication by smoking status. Figure 2 compares postoperative complications between current smoking within 4 weeks of surgery and past smoking having ceased 4 weeks or more preoperatively. Evidence from 14 547 individuals across 17 studies showed that current smoking was associated with 31% higher odds of a complication compared with past smoking (OR, 1.31 [95% CI, 1.10-1.55]). Inconsistency across the studies was modest (*I*^2^ = 43%). The findings were similar in the sensitivity analysis where the cessation threshold was flexibly defined between 4 and 8 weeks (OR, 1.35 [95% CI, 1.15-1.59]) (eFigure 1 in Supplement 1). The adjusted analyses using the multivariate logistic regression models from 7 studies (eFigure 5 in Supplement 1) also indicated elevated odds of postoperative complication among those smoking closer to surgery (adjusted OR, 1.86 [95% CI, 1.24-2.78]).

Figure 3 compares postoperative complications between current and never smoking using evidence from 9726 individuals across 14 studies. Current smoking was associated with significantly greater odds of a postoperative complication (OR, 2.83 [95% CI, 2.06-3.88]). Inconsistency across studies was high (*I*^2^ = 72%). Iteratively excluding each study and examining the *I*^2^ statistic identified Fukui, Suzuki, Matsunaga, Oh, and Takamochi^46^ and Quan, Ouyang, Zhou, Ouyang, and Xiao^77^ as large contributors to heterogeneity in the analysis. The odds of a postoperative complication among the current smoking group were slightly lower when these studies were excluded (OR, 2.75 [95% CI, 2.17-3.49]; *I*^2^ = 34%).

### Postoperative Complications by Time Quit

Focusing on shorter- and longer-term cessation, Figure 4 presents meta-analyses examining the evidence from 18 observational studies.

The meta-analysis in Figure 4A includes 5 studies comparing patients who reported smoking within 2 weeks of surgery with those who had last smoked between 2 weeks and 1 month of surgery. The estimated OR of a postoperative complication between these 2 groups was not statistically significant (OR, 1.20 [95% CI, 0.73-1.96]).

Figure 4B shows evidence across 10 studies comparing patients who reported smoking within 2 weeks of surgery with those who had last smoked between 2 weeks and 3 months before surgery. Although this analysis included information from more studies, the estimated OR remained similar (OR, 1.19 [95% CI, 0.89-1.59]).

Figure 4C compares those who reported smoking in the year before surgery with those who had stopped smoking at least 1 year prior. The estimates suggest that smoking within a year of surgery was associated with greater odds of a postoperative complication (OR, 1.13 [95% CI, 1.00-1.29]).

### Publication Bias

The funnel plots displaying possible publication bias are presented in eFigure 7 in Supplement 1. By visual inspection, there is a slight asymmetry in panel C of eFigure 7, with an absence of studies with more imprecision (in the plot’s lower left) and estimated ORs around or below 1. This indicates a possible bias among the study results with an underrepresentation of smaller scale studies reporting estimates close to or below 1 (ie, neutral or favoring shorter cessation periods over longer ones).

## Discussion

The findings from the meta-analyses suggest that for people diagnosed with cancer, smoking within 4 weeks of surgery was associated with 31% (95% CI, 9.9%-55.4%) higher odds of a postoperative complication compared with patients who have stopped smoking for at least 4 weeks prior to surgery, and 182.9% (95% CI, 106.2%-288.2%) higher odds compared with those who have never smoked. Furthermore, the adjusted analyses using the multivariate logistic regression models from 7 studies (eFigure 5 in Supplement 1) corroborate the elevated odds among those smoking closer to surgery (adjusted OR, 1.86 [95% CI, 1.24-2.78]).

The optimal time to stop smoking before surgery to minimize postoperative complications remains unclear.^81^ Interestingly, there was no statistically significant association between smoking within 2 weeks of surgery and stopping smoking between 2 weeks and 1 month, or 2 weeks to 3 months before surgery in postoperative complications, although the point estimate favored the longer cessation periods. There were lower odds of complications among people who had quit for at least 1 year compared with those still smoking within a year of surgery. These findings are based on meta-analyses of moderate to high quality, nonrandomized, observational studies with unavoidable biases from the study designs, which may compromise the reliability of the results. Furthermore, fewer studies compared shorter smoking cessation periods and possible publication bias could have led to underrepresentation of studies favoring shorter cessation periods over longer ones. Higher levels of evidence are needed to determine optimal timing of smoking cessation before cancer surgery. However, there was a paucity of randomized, smoking cessation intervention studies investigating postoperative complications in people diagnosed with cancer. The only RCT identified and included in the review was by Thomsen and colleagues,^69^ which investigated the influence of a brief smoking cessation intervention 3 to 7 days before breast cancer surgery on postoperative complications. Despite greater self-reported smoking cessation in the intervention group, postoperative complication rates were the same as in the control group (61% for both groups).

Categorizations of smoking status were often broad and inconsistent across studies. For example, the threshold for distinguishing between current and former smoking was wide-ranging: between no cessation at all until surgery^25^ to cessation up to a year before surgery.^20,26^ Furthermore, the definition of a nonsmoking group was often ambiguous, combining people who never and formerly smoked.^30,33,37,40,47,70,75,76^ Where possible, smoking status was recategorized to aid comparison in the meta-analyses using established definitions.^2^ The findings were consistent when the threshold defining current smoking was relaxed from 4 to between 4 and 8 weeks in the sensitivity analysis.

Approximately half (26 of 54) of the included studies provided no description of how smoking status was measured, and almost all of the remainder described self-report. Only 2 studies measuring smoking cessation by self-report provided details of the tool used to record smoking cessation duration.^38,39^ Shigeeda and colleagues^52^ was the only included study that biochemically verified smoking cessation by testing for cotinine using the DCT-102. Biochemical verification can increase validity compared with self-reported cessation^82,83^; therefore studies that rely on self-report could underestimate the effects of cessation on postoperative complications. Unfortunately, the findings from these 3 studies could not be included in a sensitivity analysis because the smoking cessation definitions were inconsistent (within 3 weeks of surgery, within 6 weeks of surgery, indicating yes to everyday or occasional use of cigarettes).

Collectively, the findings from this review and the robust evidence demonstrating the benefits of smoking cessation^7^ emphasize the importance of providing early and effective cessation interventions for all people undergoing surgery. For example, a recent review suggests that multicomponent smoking cessation interventions (ie, combining behavioral support and pharmacotherapy) implemented at least 4 weeks before surgery reduce the risk of complications in noncancer surgical populations.^7^ Although future RCTs can help determine the optimal timing of smoking cessation interventions in patients diagnosed with cancer, the present results suggest that smoking cessation for longer periods can maximize postoperative outcomes. Early cessation interventions therefore play an important and cost-effective role in improving the outcomes of surgical populations^84^ and should be embedded within routine cancer care. Additionally, this review identified abundant evidence of lower risks of postoperative complications among people who have never smoked,^6,17,19,20,21,25,26,33,35,36,37,38,40,41,42,44,45,48,50,53,54,55,56,57,59,61,62,64,65,66,67,68^ further highlighting the wide-reaching health and economic benefits of comprehensive, population-level tobacco control programs that discourage people from starting to smoke.

### Limitations

This study has limitations. Many studies rely on patient self-reported information to classify smoking status and cessation, which can understate smoking behavior compared with objective measures.^82,83^ This may reduce the precision or understate estimates in the included studies, and by extension, the meta-analyses. The findings from these meta-analyses could be a conservative estimate of the outcomes from recent smoking. Of note, the findings from a recent systematic review and meta-analysis of perioperative smoking cessation programs in noncancer populations (N = 13) were consistent between studies measuring exhaled carbon monoxide and studies using self-reported smoking measurements.^81^

Several studies contained information on smoking status that could not be recategorized due to unclear or unspecific language (such as *nonsmoker* without further detail)^30,33,44,66,70^ or the use of highly aggregated groups (such as smokers and nonsmokers).^31,32,33,35,37,38,41,42,43,47,53,54,61,62,68,74,75,76,79,80^ Consequently, these studies could not be included in the meta-analysis, reducing the quantity of data informing the estimate. However, by conservatively applying a uniform definition for current and past smoking, the estimates in this meta-analysis avoid inconsistent comparisons between groups by smoking status and reduce heterogeneity.

The eligibility criteria and reporting of neoadjuvant therapies varied across studies. Neoadjuvant treatments such as preoperative chemotherapy, radiation therapy, or immunotherapy may influence rates of postoperative complication, although studies have reported mixed results.^85,86,87^ Eight studies excluded patients receiving neoadjuvant therapy from their analyses.^39,48,51,53,57,68,69,72^ Where included and reported, 2% to 67% of study samples received preoperative treatment, presenting a source of between-study heterogeneity. The meta-analyses do not account for these differences across studies.

Similarly, the findings are predicated on observational data where variations in neoadjuvant therapies, smoking cessation strategies (if any), and unobserved variables could potentially confound the association between smoking status and postoperative complications. Despite this, the adjusted analyses supported the findings from the primary analysis after accounting for confounders in age, sex, and surgery characteristics. Regrettably, none of the studies informing the adjusted analyses described their approach to missing data, except Mason and colleagues,^10^ who used imputation and case deletion. The smoking cessation duration categories in this study were not readily compared with the others in the adjusted analyses.

There was moderate to high heterogeneity between the included studies, due in part to differences in cancer diagnosis and surgery type. The findings remained consistent after disaggregating the meta-analyses between lung and all other types of cancer (eFigures 3 and 4 in Supplement 1), and when omitting studies with large contributions to heterogeneity.

Additonally, some studies also collected information and defined groups by the quantity of smoking exposure with the Brinkman index or in pack-years.^35,49,56,58,59,60,74,78^ This review focuses on cessation timing, and does not examine how the quantity of smoking may contribute to postoperative complications. The choice to focus on cessation timing rather than smoking quantities reflects practical relevance to preoperative decision-making; lifetime exposure is not modifiable, whereas smoking cessation may be initiated to promote better postoperative outcomes.

## Conclusions

The results from this systematic review and meta-analysis found that longer periods of smoking cessation before surgery in patients with cancer were associated with a reduced risk of postoperative complications. Although high-quality evidence from RCTs is needed to determine the optimal timing of smoking cessation interventions to inform clinicians on the trade-offs with delaying surgery, evidence-based cessation interventions should form part of routine care for all surgical patients to maximize health and postoperative outcomes.

## Notes

Supplement 1. **eTable 1.** Eligibility Criteria **eTable 2.** Search Specification **eFigure 1.** Postoperative Complications Between Current and Past Smoking With Flexible Cutoff **eFigure 2.** Postoperative Complications Between Current and Never Smoking With Flexible Cutoff **eFigure 3.** Postoperative Complications Between Current and Past Smoking for Lung and All Other Cancers **eFigure 4.** Postoperative Complications Between Current and Never Smoking for Lung and All Other Cancers **eFigure 5.** Adjusted Estimates of Postoperative Complications Between Current and Past Smoking With Secondary Definition **eFigure 6.** 30-Day Postoperative Mortality Between Current and Past Smoking **eFigure 7.** Funnel Plot of Meta-Analyses

Supplement 2. **eTable 3. **Detailed Characteristics of Included Studies **eTable 4.** Quality Appraisal of Included Studies

Supplement 3.
Data Sharing Statement
