# Supplementary material for: Smoking and Complications After Cancer Surgery: A Systematic Review and Meta-Analysis
Source: JAMA Netw Open. 2025 Mar 7;8(3):e250295. doi: 10.1001/jamanetworkopen.2025.0295 (PMC11889474; doi:10.1001/jamanetworkopen.2025.0295)
Supplement: Supplement 1. — eTable 1. Eligibility Criteria eTable 2. Search Specification eFigure 1. Postoperative Complications Between Current and Past Smoking With Flexible Cutoff eFigure 2. Postoperative Complications Between Current and Never Smoking With Flexible Cutoff eFigure 3. Postoperative Complications Between Current and Past Smoking for Lung and All Other Cancers eFigure 4. Postoperative Complications Between Current and Never Smoking for Lung and All Other Cancers eFigure 5. Adjusted Estimates of Postoperative Complications Between Current and Past Smoking With Secondary Definition eFigure 6. 30-Day Postoperative Mortality Between Current and Past Smoking eFigure 7. Funnel Plot of Meta-Analyses [file jamanetwopen-e250295-s001.pdf]

Supplemental Online Content

Wong C, Mohamad Asfia SB, Myles PS, et al. Smoking and complications after cancer surgery: a systematic review and meta-analysis. *JAMA Netw Open.* 2025;8(3):e250295. doi:10.1001/jamanetworkopen.2025.0295

eTable 1. Eligibility Criteria

eTable 2. Search Specification

eFigure 1. Postoperative Complications Between Current and Past Smoking With Flexible Cutoff

eFigure 2. Postoperative Complications Between Current and Never Smoking With Flexible Cutoff

eFigure 3. Postoperative Complications Between Current and Past Smoking for Lung and All Other Cancers

eFigure 4. Postoperative Complications Between Current and Never Smoking for Lung and All Other Cancers

eFigure 5. Adjusted Estimates of Postoperative Complications Between Current and Past Smoking With Secondary Definition

eFigure 6. 30-Day Postoperative Mortality Between Current and Past Smoking

eFigure 7. Funnel Plot of Meta-Analyses

This supplemental material has been provided by the authors to give readers additional information about their work.

eTable 1. Eligibility Criteria

Inclusion criteria	Exclusion criteria
Adult patients* who smoke and are diagnosed with cancer from any jurisdiction undergoing any type of elective surgery	Patients undergoing emergency surgery
Outcomes measured include surgical complications, i.e. necrosis, healing delay and dehiscence, surgical site infection, wound complications, pulmonary complications, hernia, lack of fistula or bone healing, readmission or mortality, i.e. not just quit rates or abstinence	Editorials, comment or discussion papers, qualitative studies, reviews, economic evaluations, case reports, case series, book chapters and conference articles or abstracts.
Observational studies (case-control, cohort, cross-sectional)	Postoperative complications not reported.
Randomized Controlled Trials	For interventional studies, details of smoking cessation intervention not provided, e.g. brief advice plus nicotine replacement therapy (NRT) or pharmacotherapy where the type of NRT or pharmacotherapy is not described.
For interventional studies, all smoking cessation interventions, including brief advice, behavioural support, pharmacotherapy [nicotine replacement therapy, varenicline, bupropion], individually or in combination, commenced at least one week before surgery.	Case reports
All comparators	Only report outcomes beyond 12 months
Report outcomes at 12 months or less	

eTable 2. Search specification

Search No.	Terms
1	smoking:ti,ab
2	smoker*:ti,ab
3	smoking'/de OR 'smoking reduction'/de OR 'smoking cessation'/de OR 'smoking cessation agent'/de
4	#1 OR #2 OR #3
5	tumo?r*:ti,ab
6	neoplasm*:ti,ab
7	cancer:ti,ab
8	malign*:ti,ab
9	oncolog*:ti,ab
10	carcinoma*:ti,ab
11	neoplasm'/de
12	#5 OR #6 OR #7 OR #8 OR #9 OR #10 OR #11
13	complication*:ti,ab
14	postop*:ti,ab
15	post op*':ti,ab
16	periop*:ti,ab
17	peri op*':ti,ab
18	preop*:ti,ab
19	pre op*':ti,ab
20	presurg*:ti,ab
21	pre surg*':ti,ab
22	intraop*:ti,ab
23	intra op*':ti,ab
24	surg*:ti,ab
25	anaesthe*:ti,ab
26	anesthe*:ti,ab
27	elective:ti,ab
28	preanesthe*:ti,ab
29	pre anesthe*':ti,ab
30	preanaesthe*:ti,ab
31	pre anaesthe*':ti,ab
32	perisurg*:ti,ab
33	peri surg*':ti,ab
34	preadmi*:ti,ab
35	pre admi*':ti,ab
36	preoperative care'/de OR 'postoperative care'/de OR 'perioperative period'/de OR 'postoperative period'/de OR 'intraoperative period'/de OR 'preoperative period'/de OR 'elective surgery'/de OR 'postoperative complication'/de
37	#13 OR #14 OR #15 OR #16 OR #17 OR #18 OR #18 OR #19 OR #20 OR #21 OR #22 OR #23 OR #24 OR #25 OR #26 OR #27 OR #28 OR #29 OR #30 OR #31 OR #32 OR #33 OR #34 OR #35 OR #36
38	#4 AND #12 AND #37

eTable 3: Detailed Characteristics of Included Studies

[Refer to Supplementary eTable 3 within Excel document]

eTable 4: Quality Appraisal of Included Studies

[Refer to Supplementary eTable 4 within Excel document]

eFigure 1: Postoperative complications between current and past smoking with flexible cutoff

Study or Subgroup	Current Events	Current Total	Past Events	Past Total	Weight	Odds Ratio IV, Random, 95% CI
Al Natour et al,[29] 2021	25	467	59	1480	5.6%	1.36 [0.84, 2.20]
Barrera et al,[31] 2005	12	52	34	184	3.3%	1.32 [0.63, 2.79]
Bayley et al,[9] 2022	61	196	208	842	7.4%	1.38 [0.98, 1.94]
Chang et al,[45] 2000	51	90	53	150	5.0%	2.39 [1.40, 4.09]
Fukui et al,[46] 2019	22	72	52	313	4.5%	2.21 [1.23, 3.96]
Goodwin et al,[71] 2005	21	54	31	78	3.6%	0.96 [0.47, 1.96]
Groth et al,[36] 2009	13	39	35	81	3.0%	0.66 [0.30, 1.46]
Inoue et al,[48] 2020	21	76	36	189	4.2%	1.62 [0.87, 3.02]
Jung et al,[72] 2015	36	247	28	275	5.1%	1.51 [0.89, 2.55]
Kadomatsu et al,[49] 2022	20	88	130	547	5.0%	0.94 [0.55, 1.61]
Kamarajah et al,[73] 2021	206	282	349	530	7.7%	1.41 [1.02, 1.93]
Kozub et al,[63] 2019	20	67	43	101	3.9%	0.57 [0.30, 1.11]
Kuri et al,[50] 2005 (1)	74	82	39	66	2.6%	6.40 [2.66, 15.43]
Lugg et al,[64] 2017	30	166	29	245	4.8%	1.64 [0.94, 2.86]
Mason et al,[10] 2009	135	1992	294	4949	9.2%	1.15 [0.93, 1.42]
Mediratta et al,[65] 2016	69	852	110	1587	7.7%	1.18 [0.87, 1.62]
Quan et al,[77] 2019	121	863	29	193	6.1%	0.92 [0.59, 1.43]
Seok et al,[67] 2014	27	110	21	122	4.1%	1.56 [0.83, 2.97]
Yamamichi et al,[57] 2021	10	117	33	819	3.4%	2.23 [1.07, 4.65]
Yoshida et al,[58] 2016	20	43	63	142	3.7%	1.09 [0.55, 2.16]
**Total (95% CI)**		**5955**		**12893**	**100.0%**	**1.35 [1.15, 1.59]**
Total events	994		1676			

Heterogeneity: Tau² = 0.06; Chi² = 40.11, df = 19 (P = 0.003); I² = 53%
Test for overall effect: Z = 3.61 (P = 0.0003)

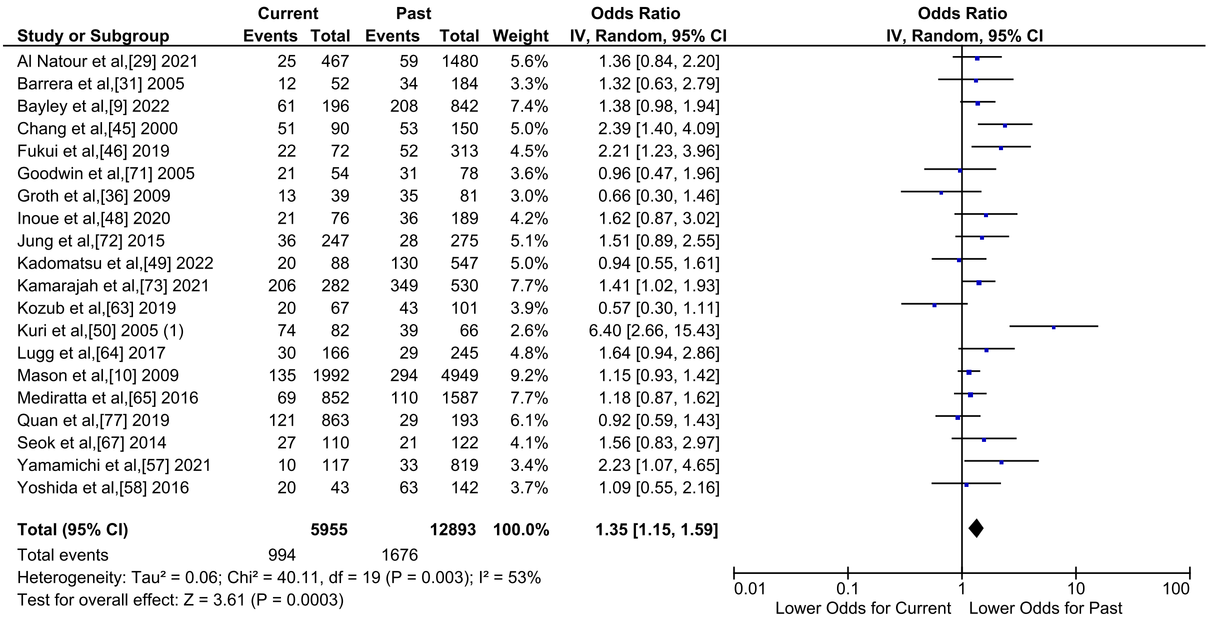

Footnotes
(1) Maximum single complication type

Note: Unadjusted odds ratio of any postoperative complication between Current (last smoked within four to eight weeks before surgery) and Past (last smoked beyond four to eight weeks before surgery) smoking. Counts of events/totals are larger for Barrera et al,[31] 2005; Kuri et al,[50] 2005; and Lugg et al,[64] 2017 than under the strict four-week definition since they contain groupings that fit the relaxed definition; IV: inverse variance.

eFigure 2: Postoperative complications between current and never smoking with flexible cutoff

	Current		Never			Odds Ratio
Study or Subgroup	Events	Total	Events	Total	Weight	IV, Random, 95% CI
Al Natour et al,[29] 2021	89	467	27	320	7.8%	2.56 [1.62, 4.03]
Barrera et al,[31] 2005	12	52	5	64	4.3%	3.54 [1.16, 10.83]
Chang et al,[45] 2000	51	90	176	478	7.8%	2.24 [1.42, 3.54]
Fukui et al,[46] 2019	22	72	9	256	5.7%	12.08 [5.25, 27.78]
Goodwin et al,[71] 2005	20	54	58	383	6.9%	3.30 [1.77, 6.12]
Inoue et al,[48] 2020	21	76	17	290	6.4%	6.13 [3.04, 12.37]
Jung et al,[72] 2015	36	247	42	813	7.7%	3.13 [1.96, 5.01]
Kadomatsu et al,[49] 2022	20	88	31	276	6.8%	2.32 [1.25, 4.33]
Kamarajah et al,[73] 2021	206	282	220	356	8.4%	1.68 [1.19, 2.35]
Kozub et al,[63] 2019	20	67	5	12	3.8%	0.60 [0.17, 2.10]
Kuri et al,[50] 2005 (1)	74	82	19	40	5.0%	10.22 [3.92, 26.64]
Lugg et al,[64] 2017	30	166	1	51	1.9%	11.03 [1.47, 83.02]
Mason et al,[10] 2009	135	1992	27	1024	8.0%	2.68 [1.76, 4.09]
Quan et al,[77] 2019	121	863	143	1413	8.8%	1.45 [1.12, 1.88]
Yamamichi et al,[57] 2021	10	117	7	815	4.9%	10.79 [4.02, 28.94]
Yoshida et al,[58] 2016	20	43	21	61	5.8%	1.66 [0.75, 3.68]
**Total (95% CI)**		**4758**		**6652**	**100.0%**	**3.06 [2.23, 4.20]**
Total events	887		808			

Heterogeneity: Tau² = 0.28; Chi² = 68.70, df = 15 (P < 0.00001); I² = 78%
Test for overall effect: Z = 6.94 (P < 0.00001)

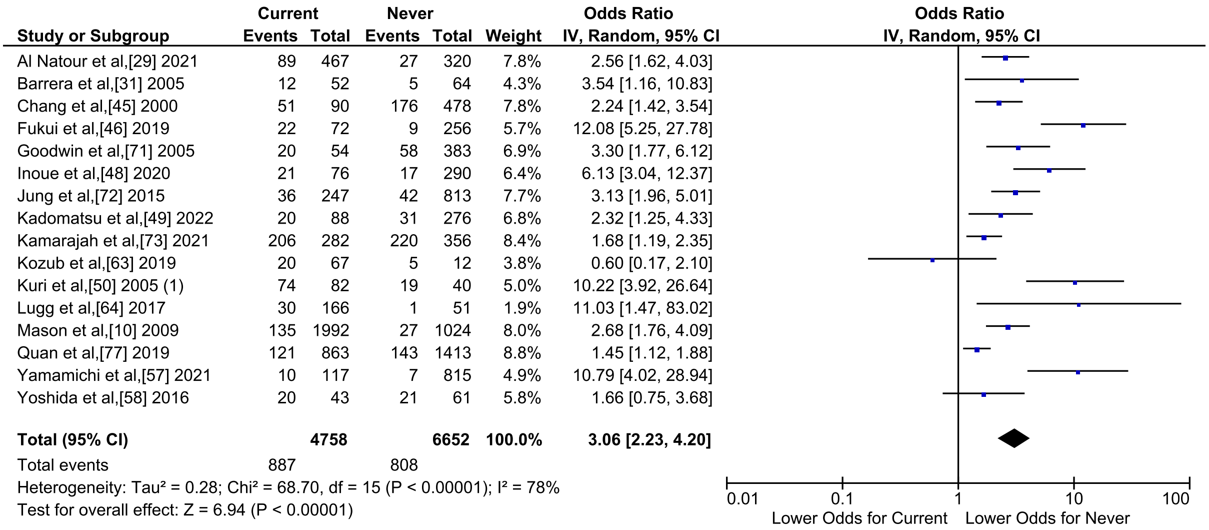

Footnotes
(1) Maximum single complication type

Note: Unadjusted odds ratio of any postoperative complication between Current (last smoked within four to eight weeks before surgery) and Never smoking. Counts of events/totals are larger for Barrera et al,[31] 2005; Kuri et al,[50] 2005; and Lugg et al,[64] 2017 than under the strict four-week definition since they contain groupings that fit the relaxed definition; IV: inverse variance.

eFigure 3: Postoperative complications between current and past smoking for lung and all other cancers

Study or Subgroup	Current Events	Current Total	Past Events	Past Total	Weight	Odds Ratio IV, Fixed, 95% CI
**4.8.1 Lung Cancer**						
Al Natour et al,[29] 2021	25	467	59	1480	6.0%	1.36 [0.84, 2.20]
Barrera et al,[31] 2005	3	13	34	184	0.8%	1.32 [0.35, 5.07]
Bayley et al,[9] 2022	61	196	208	842	11.9%	1.38 [0.98, 1.94]
Fukui et al,[46] 2019	22	72	52	313	4.1%	2.21 [1.23, 3.96]
Groth et al,[36] 2009	13	39	35	81	2.2%	0.66 [0.30, 1.46]
Kadomatsu et al,[49] 2022	20	88	130	547	4.8%	0.94 [0.55, 1.61]
Kozub et al,[63] 2019	20	67	43	101	3.2%	0.57 [0.30, 1.11]
Lugg et al,[64] 2017	24	111	29	245	3.9%	2.05 [1.13, 3.73]
Mason et al,[10] 2009	135	1992	294	4949	31.1%	1.15 [0.93, 1.42]
Seok et al,[67] 2014	27	110	21	122	3.4%	1.56 [0.83, 2.97]
**Subtotal (95% CI)**		**3155**		**8864**	**71.4%**	**1.23 [1.07, 1.41]**
Total events	350		905			
Heterogeneity: Chi² = 16.77, df = 9 (P = 0.05); I² = 46%						
Test for overall effect: Z = 2.93 (P = 0.003)						
**4.8.2 All Other Cancers**						
Chang et al,[45] 2000	51	90	53	150	4.8%	2.39 [1.40, 4.09]
Goodwin et al,[71] 2005	21	54	31	78	2.7%	0.96 [0.47, 1.96]
Inoue et al,[48] 2020	21	76	36	189	3.6%	1.62 [0.87, 3.02]
Jung et al,[72] 2015	36	247	28	275	5.0%	1.51 [0.89, 2.55]
Kuri et al,[50] 2005	47	62	39	66	2.4%	2.17 [1.01, 4.64]
Quan et al,[77] 2019	121	863	29	193	7.2%	0.92 [0.59, 1.43]
Yoshida et al,[58] 2016	20	43	63	142	2.9%	1.09 [0.55, 2.16]
**Subtotal (95% CI)**		**1435**		**1093**	**28.6%**	**1.39 [1.12, 1.73]**
Total events	317		279			
Heterogeneity: Chi² = 10.46, df = 6 (P = 0.11); I² = 43%						
Test for overall effect: Z = 2.93 (P = 0.003)						
**Total (95% CI)**		**4590**		**9957**	**100.0%**	**1.27 [1.13, 1.43]**
Total events	667		1184			
Heterogeneity: Chi² = 28.06, df = 16 (P = 0.03); I² = 43%						
Test for overall effect: Z = 4.04 (P < 0.0001)						
Test for subgroup differences: Chi² = 0.83, df = 1 (P = 0.36), I² = 0%						

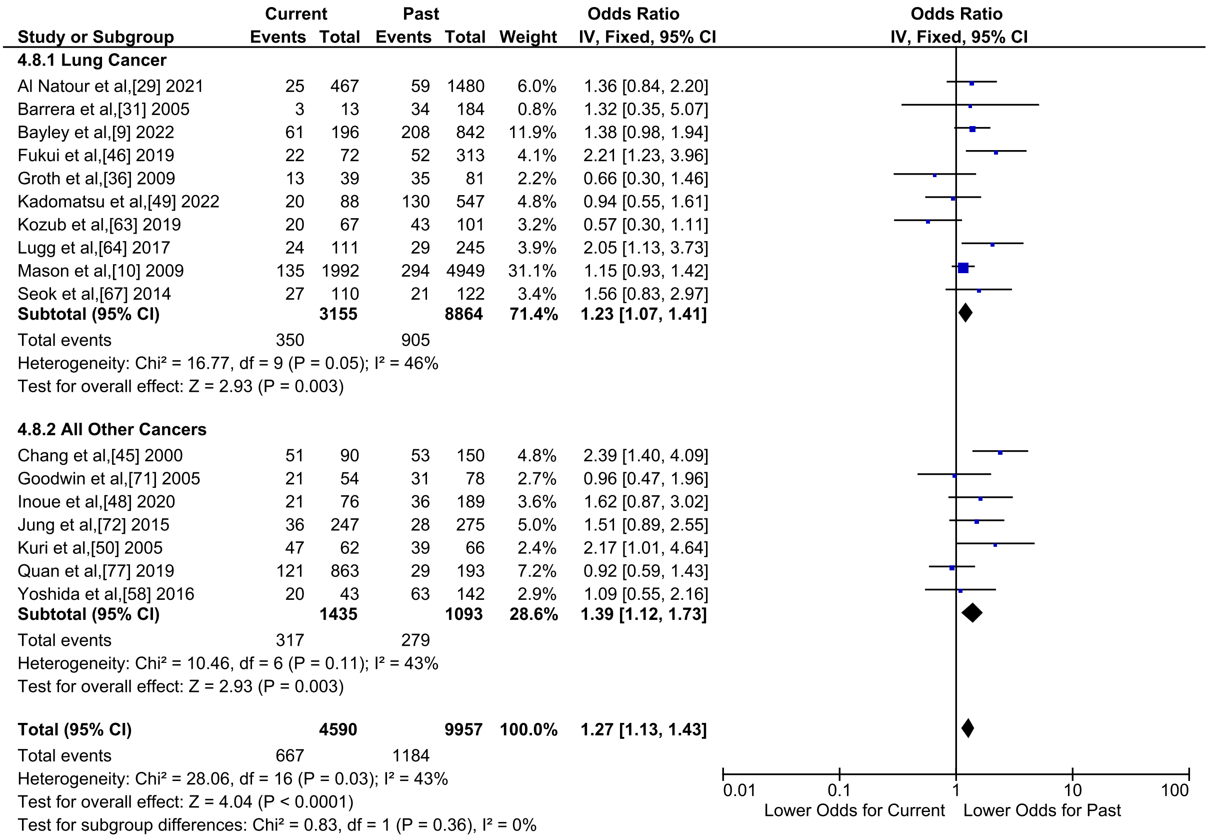

Note: Unadjusted odds ratio of any postoperative complication between Current (last smoked within four weeks before surgery) and Past (last smoked beyond four weeks before surgery) smoking. Studies grouped by lung cancer and all other cancers. IV: inverse variance.

eFigure 4: Postoperative complications between current and never smoking for lung and all other cancers

Study or Subgroup	Current Events	Current Total	Never Events	Never Total	Weight	Odds Ratio IV, Fixed, 95% CI
**4.9.1 Lung Cancer**						
Al Natour et al,[29] 2021	89	467	27	320	10.1%	2.56 [1.62, 4.03]
Barrera et al,[31] 2005	3	13	5	64	0.8%	3.54 [0.73, 17.20]
Fukui et al,[46] 2019	22	72	9	256	3.0%	12.08 [5.25, 27.78]
Kadomatsu et al,[49] 2022	20	88	31	276	5.4%	2.32 [1.25, 4.33]
Kozub et al,[63] 2019	20	67	5	12	1.3%	0.60 [0.17, 2.10]
Lugg et al,[64] 2017	24	111	1	51	0.5%	13.79 [1.81, 105.06]
Mason et al,[10] 2009	135	1992	27	1024	11.9%	2.68 [1.76, 4.09]
Quan et al,[77] 2019	121	863	143	1413	31.4%	1.45 [1.12, 1.88]
**Subtotal (95% CI)**		**3673**		**3416**	**64.5%**	**2.06 [1.72, 2.47]**
Total events	434		248			
Heterogeneity: Chi² = 34.51, df = 7 (P < 0.0001); I² = 80%						
Test for overall effect: Z = 7.85 (P < 0.00001)						
**4.9.2 All Other Cancers**						
Chang et al,[45] 2000	51	90	176	478	10.1%	2.24 [1.42, 3.54]
Goodwin et al,[71] 2005	20	54	58	383	5.5%	3.30 [1.77, 6.12]
Inoue et al,[48] 2020	21	76	17	290	4.3%	6.13 [3.04, 12.37]
Jung et al,[72] 2015	36	247	42	813	9.5%	3.13 [1.96, 5.01]
Kuri et al,[50] 2005	47	62	19	40	2.9%	3.46 [1.48, 8.10]
Yoshida et al,[58] 2016	20	43	21	61	3.3%	1.66 [0.75, 3.68]
**Subtotal (95% CI)**		**572**		**2065**	**35.5%**	**2.96 [2.32, 3.77]**
Total events	195		333			
Heterogeneity: Chi² = 7.88, df = 5 (P = 0.16); I² = 37%						
Test for overall effect: Z = 8.74 (P < 0.00001)						
**Total (95% CI)**		**4245**		**5481**	**100.0%**	**2.34 [2.03, 2.71]**
Total events	629		581			
Heterogeneity: Chi² = 47.87, df = 13 (P < 0.00001); I² = 73%						
Test for overall effect: Z = 11.52 (P < 0.00001)						
Test for subgroup differences: Chi² = 5.47, df = 1 (P = 0.02), I² = 81.7%						

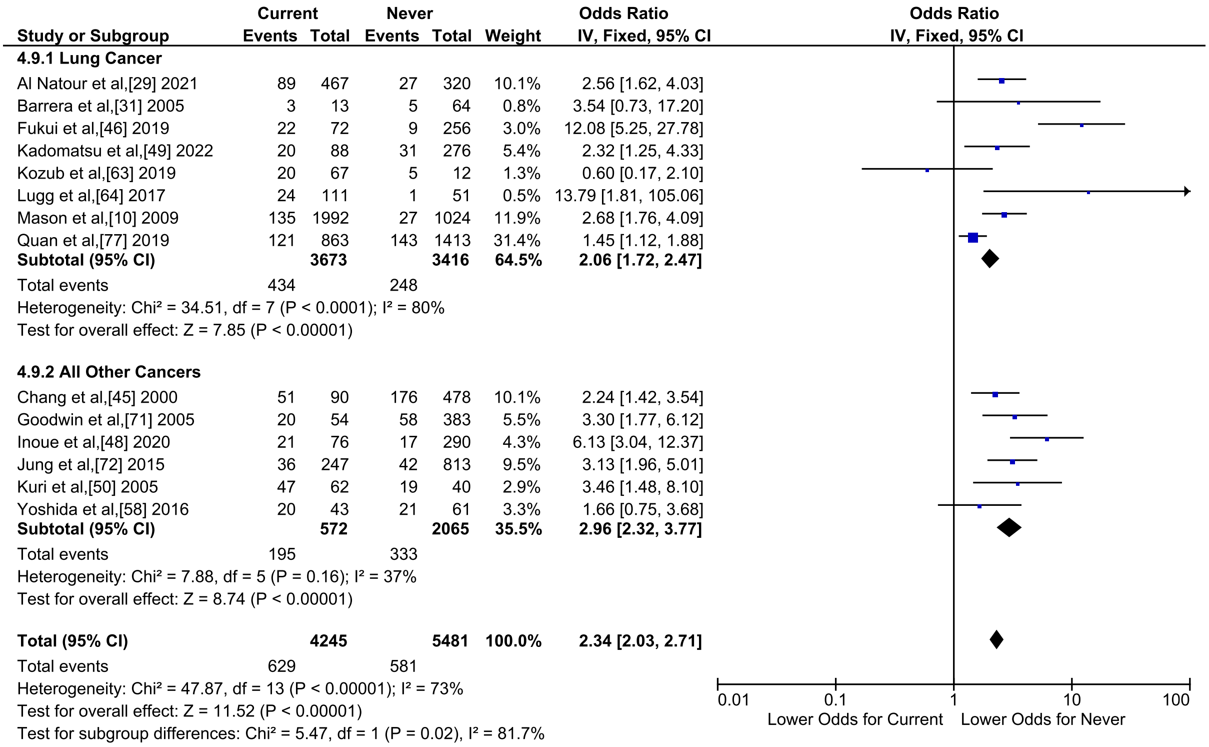

Note: Unadjusted odds ratio of any postoperative complication between Current (last smoked within four weeks before surgery) and Never smoking. Studies grouped by lung cancer and all other cancers. IV: inverse variance.

eFigure 5: Adjusted estimates of postoperative complications between current and past smoking with secondary definition

Study or Subgroup	log[Odds Ratio]	SE	Weight	Odds Ratio IV, Random, 95% CI	Odds Ratio IV, Random, 95% CI
Bayley et al,[9] 2022	0.0275	0.2818	16.5%	1.03 [0.59, 1.79]	
Kadomatsu et al,[49] 2022	0.2772	0.3382	14.5%	1.32 [0.68, 2.56]	
Kuri et al,[50] 2005	1.772	0.6434	7.1%	5.88 [1.67, 20.76]	
Napolitano et al,[40] 2021	0.2517	0.16	20.9%	1.29 [0.94, 1.76]	
Seok et al,[67] 2014	0.6962	0.3583	13.8%	2.01 [0.99, 4.05]	
Shigeeda et al,[52] 2023	1.5162	0.383	13.0%	4.55 [2.15, 9.65]	
Yamamichi et al,[57] 2021	0.7178	0.3462	14.2%	2.05 [1.04, 4.04]	
**Total (95% CI)**			**100.0%**	**1.86 [1.24, 2.78]**	

Heterogeneity: $Tau^2 = 0.18$; $Chi^2 = 17.09$, df = 6 (P = 0.009); $I^2 = 65\%$

Test for overall effect: Z = 3.02 (P = 0.003)

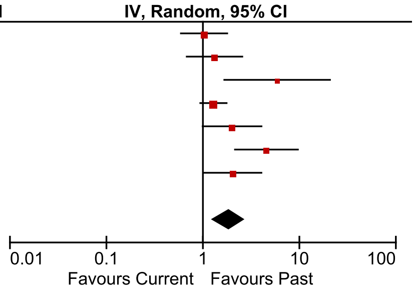

Note: Adjusted odds ratio of any postoperative complication by period between Current (last smoked within four weeks before surgery) and Past (last smoked beyond four weeks before surgery) smoking. Studies adjusted for age, sex, comorbidities such as diabetes or COPD, and surgery characteristics. SE: standard error; IV: inverse variance.

eFigure 6: 30-day postoperative mortality between current and past smoking

Study or Subgroup	Current Events	Current Total	Past Events	Past Total	Weight	Odds Ratio M-H, Random, 95% CI
Delman et al,[34] 2021	2	236	7	365	7.3%	0.44 [0.09, 2.12]
Kamarajah et al,[73] 2021	24	282	24	530	14.1%	1.96 [1.09, 3.52]
Lugg et al,[64] 2017	2	166	6	245	7.2%	0.49 [0.10, 2.44]
Mason et al,[10] 2009	31	1999	74	4966	15.2%	1.04 [0.68, 1.59]
Mediratta et al,[65] 2016	22	852	78	1587	14.9%	0.51 [0.32, 0.83]
Napolitano et al,[40] 2021	11	572	12	1144	12.4%	1.85 [0.81, 4.22]
Quan et al,[77] 2019	5	863	0	193	3.2%	2.48 [0.14, 45.03]
Shigeeda et al,[52] 2023	4	41	4	736	8.2%	19.78 [4.76, 82.23]
Xu et al,[76] 2020	6	267	10	282	10.8%	0.63 [0.22, 1.74]
Yoshida et al,[58] 2016	1	43	1	142	3.4%	3.36 [0.21, 54.83]
Yoshida et al,[59] 2018	1	42	1	156	3.4%	3.78 [0.23, 61.74]
**Total (95% CI)**		**5363**		**10346**	**100.0%**	**1.31 [0.74, 2.33]**
Total events	109		217			

Heterogeneity: Tau² = 0.52; Chi² = 36.22, df = 10 (P < 0.0001); I² = 72%
Test for overall effect: Z = 0.93 (P = 0.35)

Odds Ratio
M-H, Random, 95% CI

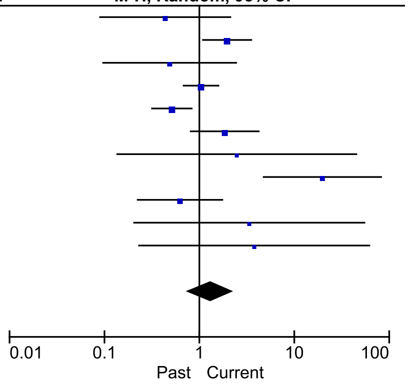

Note: Unadjusted odds ratio of mortality within 30 days between Current (last smoked within four weeks before surgery) and Past (last smoked beyond four weeks before surgery) smoking. M-H: Mantel-Haenszel.

eFigure 7: Funnel plot of meta-analyses

(a) Current v Past

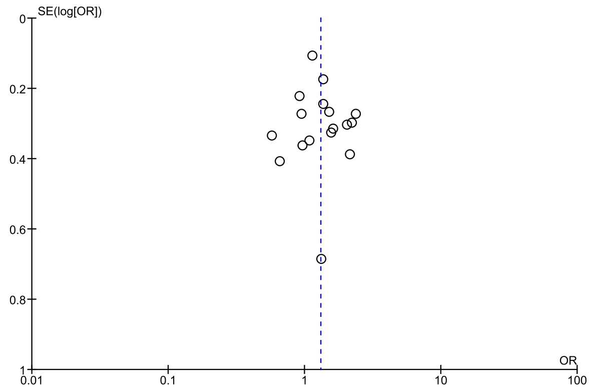

(b) Current v Never

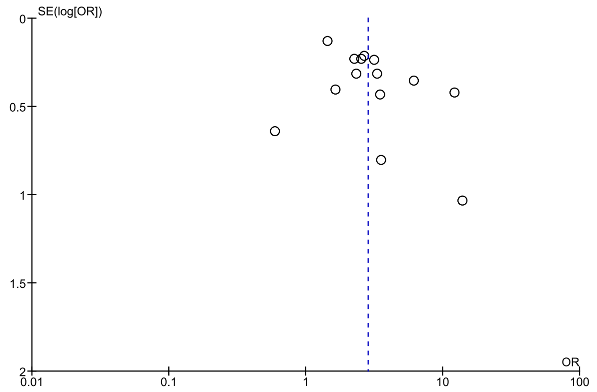

(c) By Cessation Duration

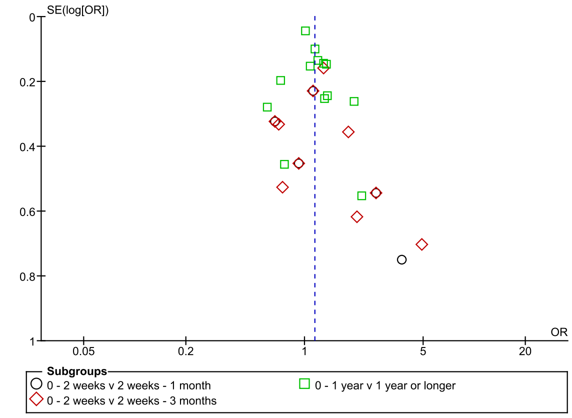

Note: Plot of study estimates and study size for (a) Figure 2 (b) Figure 3 and (c) Figure 4; SE: standard error; OR: odds ratio.
