# Supplementary material for: Smoking and Complications After Cancer Surgery: A Systematic Review and Meta-Analysis
Source: JAMA Netw Open. 2025 Mar 7;8(3):e250295. doi: 10.1001/jamanetworkopen.2025.0295 (PMC11889474; doi:10.1001/jamanetworkopen.2025.0295)
Supplement: Supplement 3. — Data Sharing Statement [file jamanetwopen-e250295-s003.pdf]

Data Sharing Statement

Wong. Smoking and Complications After Cancer Surgery. *JAMA Netw Open*. Published March 07, 2025. doi:10.1001/jamanetworkopen.2025.0295

Data
Data available: No

Additional Information
Explanation for why data not available: The data for the meta-analysis are contained in published studies. Relevant information about the studies included for the systematic review are contained in the supplement files.
